# Cervical Intraepithelial Neoplasia Grade 3 (CIN3) in Women Younger than 30 Years Was Significantly Associated with HPV16/18 Genotypes

**DOI:** 10.3390/cancers16112043

**Published:** 2024-05-28

**Authors:** Maria Teresa Bruno, Marco Marzio Panella, Gaetano Valenti, Zaira Ruggeri, Francesco Sgalambro, Salvatore Reina, Liliana Mereu

**Affiliations:** 1 Gynecology and Obstetrics Unit, Department of General Surgery and Medical-Surgical Specialty, Rodolico University Hospital, University of Catania, 95124 Catania, Italy; mpanella@unict.it (M.M.P.); f.sgalambro@policlinico.unict.it (F.S.); salvatore.reina@studium.unict.it (S.R.); liliana.mereu@unict.it (L.M.); 2 Multidisciplinary Research Center in Papillomavirus Pathology, Chirmed, University of Catania, 95123 Catania, Italy; gaetano.valenti@humanitascatania.it; 3 Humanitas, Gynaecologic Oncology Unit, 95045 Catania, Italy; 4 Cervical Cancer Screening Unit, Level II, ASP Messina, 98123 Messina, Italy; zaira.ruggeri@asp.messina.it

**Keywords:** CIN3, genotype, HPV 16/18, no 16/18 HPV, age

## Abstract

**Simple Summary:**

The present study aimed to evaluate the age-related distribution of HPV 16/18 genotypes and non-16/18 HPV genotypes in unvaccinated women. CIN3 in women younger than 30 years was significantly associated with HPV16/18 genotypes. The surprising fact of the present study is represented by the fact that in women under the age of 30, almost 90% of CIN3 cases were associated with HPV16/18, while CIN3 with non-16/18 HPV genotypes develops slowly and in older age. The data from the present study suggest that the risk of CIN3 is related to the woman’s age and hr HPV genotype. These data are essential to optimize current and future screening programs.

**Abstract:**

Background. The objective of the present study is to investigate the age-specific distribution of HPV genotypes in CIN3 lesions in screened unvaccinated women. These data are essential to optimize current and future screening programs. Methods. A multicenter retrospective study was conducted. A total of 408 unvaccinated women with positive histology and a high-risk HPV genotype were enrolled. Each woman at baseline had HPV DNA testing and HPV genotyping, and all women underwent targeted biopsy and/or treatment with a loop electrosurgical excision procedure (LEEP) before entering the study. We divided the genotypes into HPV16/18 and HPV non-16/18 (HPV31/33/45/35/39/51/52/58/59/66/68). Women were divided into increasing age categories: <30, 30–44, and ≥45. Results. The percentage of CIN3 associated with HPV16/18 is maximum in women under 30 years of age (85.1%), drops to 75.6% in women aged between 30 and 44 years, and up to 47.2% in women over 45 years. CIN3 in women younger than 30 years was significantly associated with HPV16/18 genotypes (*p* = 0). Discussion. The data from the present study suggest that the risk of CIN3 is related to the woman’s age and hr HPV genotype. The data highlight two different types of CIN3: a more frequent type, related to HPV16/18, which develops rapidly and in young women, and another, relating to non-16/18 HPV, which develops later at an advanced age and slowly, through low-grade lesions.

## 1. Introduction

The prevention of cervical squamous cell carcinoma (CC) involves the early diagnosis and treatment of preneoplastic lesions of the cervix. CIN3 is the preneoplastic lesion of cervical cancer and is the endpoint targeted by cervical cancer screening programs and in HPV vaccine trials to evaluate the effectiveness of primary and secondary prevention strategies. Although HPV screening and vaccines have been shown to be effective [1,2], high-grade cervical intraepithelial neoplasia (CIN3) is still a problem, even in high-resource areas [3]. The natural history of cervical cancer shows how the progression from HPV infection to the persistence of the virus, development of CIN3, and finally to invasive cervical cancer appears to take, on average, up to about 15 years, although cases of tumors do occur in rapid onset [4,5,6]. Follow-up data from meta-analyses show that without treatment, 15–40% or more of all CIN3 lesions will naturally regress in immunocompetent women [7,8,9,10]. On the other hand, previous studies have shown that only 2 years of HPV 16 infection is enough for the appearance of CIN3. Furthermore, Ronco et al. [11] reported a three-fold higher histologically confirmed CIN3 detection rate in women aged 25–35 years compared to women aged 35–65 years. There is evidence that HPV16/18-related cervical cancers occur on average at a younger age than cancers due to other HPV genotypes [12,13]. This may be due to the fact that genotypes 16 and 18 are more commonly integrated into the host genome, while other genotypes, such as HPV31 and 33, are more likely to be episomal. Furthermore, it may also be related to the sexual behavior of young women and their sexual partners.

Persistent hr HPV infection is a prerequisite for the development of cervical intraepithelial neoplasia grade 3 (CIN3). In the most recent evaluation by the International Agency for Research on Cancer (IARC), 12 genotypes (HPV16, 18, 31, 33, 35, 39, 45, 51, 52, 56, 58, and 59) were classified as carcinogenic and HPV 68 and 66 have been classified as probably carcinogenic to humans (group 2A). Therefore, several HPV types have been characterized as oncogenic but not all have the same carcinogenic potential; HPV16 in particular has shown unique carcinogenic potential. The genotypes that confer greater persistence with progression are 16, 31, and 33 very frequent in Italy, while HPV 16, 33, 31, and 18 are more frequent in the United States [6,14]. Since cervical precancerous lesions are the target of cervical cancer screening programs, it is important to better understand the association between age, CIN3, and HPV genotype. Vaccines currently in use protect women against HPV 16 and 18. If a younger age at diagnosis of precancerous lesions is associated with HPV16, future cervical cancer screening programs could begin at an older age in women vaccinated against HPV. The objective of the present study is to investigate the age-specific distribution of genotypes in CIN3 lesions in screened unvaccinated women. 

## 2. Materials and Methods

A multicenter retrospective study was conducted in which three second-level centers for the diagnosis and treatment of HPV lesions and cervical cancer took part: Gynecology and Obstetrics Operating Unit, Rodolico University Hospital, Department of General Surgery and Medical- Surgical Specialty of the University of Catania; Gynecological Oncology Unit, Humanitas Hospital; and the second-level screening center of the ASP of Messina. Data from patients referred for colposcopy from January 2017 to December 2020 due to a positive screening smear or immediately before excisional treatment were collected in a dedicated database. Inclusion criteria were unvaccinated women with positive histology and high-risk HPV genotype.

Women who did not have HPV test results, who were vaccinated, or who had a history of cervical cancer were excluded. A ThinPrep PreservCyt cervical specimen (Hologic Inc., Bedford, MA, USA) was collected in all patients at baseline visits to perform HPV DNA testing and HPV genotyping. All women underwent targeted biopsy and/or loop electrosurgical excision procedure (LEEP) treatment before starting the study.

All included centers used the same molecular technique for HPV DNA detection and genotyping.

### 2.1. HPV DNA Testing and HPV Genotyping

Exocervical cytology samples were collected and placed in Thin Prep solution. The samples were sent to the laboratory for DNA extraction and genotyping of the viral DNA via genetic amplification followed by hybridization with genotype-specific probes capable of identifying the majority of HPV genotypes of the genital region, high-density HPV genotypes risk (16, 18, 26, 31, 33, 35, 39, 45, 51, 52, 53, 56, 58, 59, 66, 68, 73, 82), low risk (6, 11, 40, 43, 44, 54, 70), and indefinite risk (69, 71, 74)]. The commercial method used was the MAG NucliSenseasy system (bioMerieux SA, Marct l‘Etoile, France). HPV testing was performed using a previously reported method [15]. We divided the genotypes into HPV16/18 and non-HPV16/HPV18 hr HPV genotypes (HPV31/33/45/35/39/51/52/58/59/66/68).

In cases of multiple infections, for the correct attribution of a genotype to the lesion when the genotypes detected are multiple, we used the HPV E6/E7 mRNA test, attributing the infection to the genotype indicated with the positive test.

Women were divided into increasing age categories: <30, 30–44, and ≥45. The <30 years of age group is made up of 134 women and the age group between 30 and 44 years is made up of 206 women. The >45 age group consists of 68 women.

For histological diagnosis, the WHO classification was followed.

The ethics committee of the University Hospital (Catania 2) waived the obligation of ethical approval and informed consent because the study used previously archived data, according to current legislation (20 March 2008) (AIFA). According to Italian law, patient consent is not mandatory in a retrospective study.

### 2.2. Statistical Analysis

Statistical analysis was performed using the SPSS software package for Windows (version 15.0, SPSS, Chicago, IL, USA). Descriptive statistics are expressed as frequency, arithmetic mean, and percentages. The results are summarized in tables. The relationship between categorical variables was assessed using Chi-square tests or Fisher exact tests, depending on the sample size. A logistic regression analysis was performed to study the association between age and HPV16 positive status used as predictive variables. This was performed for CIN3 with HPV16 as the categorical outcome and age was treated as a continuous variable. Their association was assessed by estimating odds ratios (ORs) with 95% confidence intervals (CIs). *p* < 0.05 was considered statistically significant.

## 3. Results

**Figure 1:**
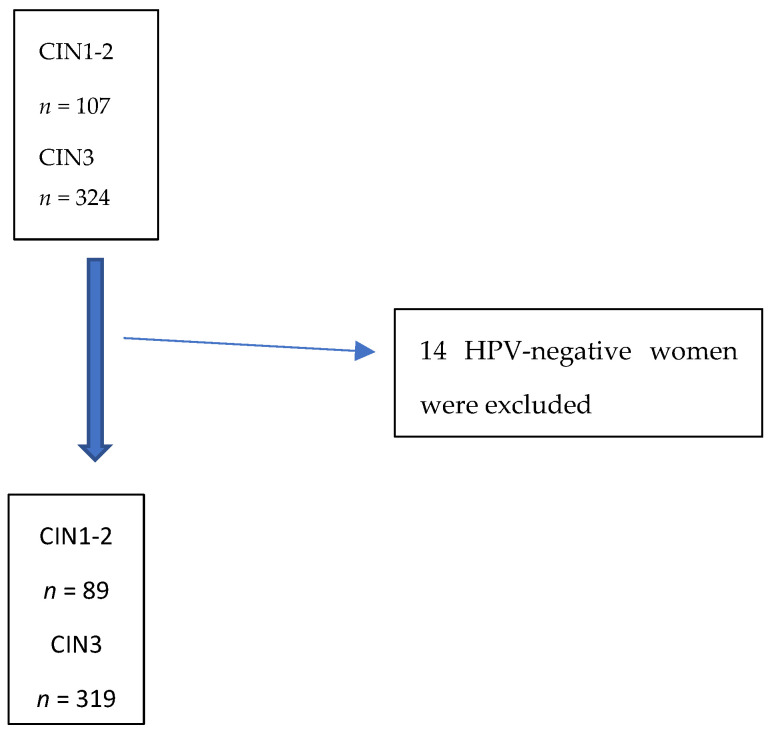
Flowchart of the study population.

The study period included 324 consecutive unvaccinated women with CIN3 and 107 women with CIN1 and CIN2 on histology. In the analysis, we included only HPV-positive samples; 14 (4%) hr HPV-negative cases were excluded. In the end, the study sample was represented by 408 women: 319 cases of CIN3, 17 cases of CIN2, and 72 cases of CIN1 (Figure 1). Before the start of the study, 319 cases of CIN3, 8 cases of CIN2, and 5 cases of CIN1 were subjected to LEEP; 9 cases of CIN2 underwent conservative therapy, while 67 cases of CIN1 underwent follow-up [16].

The women enrolled have an average age of 36.3 years (a range of 17–72 years). There are 89 cases of women with CIN1 and CIN2, 27 with HPV 16/18 genotype, and 62 with non-16/18 HPV genotype.

The study sample with CIN3 consists of 319 women: 235 cases with HPV16/18 genotype and 84 with non-16/18 HPV genotype (Table 1).

Overall, the most represented genotype is HPV16 with 252/408 cases (61.7%), followed by genotypes 31 with 39 cases (8.8%), 33 with 29 cases (7.1%), and 52 with 13 cases (3.18%). Genotype 18, with three cases (0.7%), is poorly represented in the present study.

The HPV16/18 group consists of 235 CIN3 cases, with an average age of 34.1 (a range of 17–51). The most represented genotype is HPV 16 with 232 (98.7%) positive cases of CIN3 HPV 16, while genotype 18 is quite rare in the present study with only 3 positive cases of CIN3 HPV 18.

The non-16/18 HPV group was made up of 84 cases (26.3%) of CIN3. They are represented by 12 high-risk genotypes. The most represented genotypes are HPV31 with 36 cases (42.8%), HPV33 with 14 (16.6%) cases, HPV52 with 8 cases, and genotypes 45 and 51 with 5 cases. The population has an average age of 54.8 years (a range of 26–72) (Table 2).

In women <30 years old, 85.1% (80/94) of CIN2 and CIN3 cases were associated with HPV16/18. In women aged 30 to 44 years, 75.6% (130/172) of cases were HPV16-positive; in women aged >45 years, 47.2% (25/53) were HPV16-positive. On the other hand, only 14.9% of CIN3 in women under 30 were positive for non-HPV16/18 genotypes, 42% of women aged between 30 and 44 years were, and 28% of women aged >45 years were (Table 3).

The association between CIN3 and HPV16/18 in the study population shows a significant decrease with increasing age. In contrast, it is interesting to note that non-16/18 HPV genotypes become increasingly prevalent with advancing age. The percentage of CIN3 associated with HPV16/18 is at its maximum in women under 30 years of age (85.1%), decreases to 75.6% in women aged between 30 and 44 years, and is up to 47.2% in women over 45 years. CIN3 in women younger than 30 years was significantly associated with HPV16/18 genotypes (*p* = 0) (Table 4).

To further explore the relationship between age and HPV, a logistic regression analysis was performed. Young age upon diagnosis was strongly associated with HPV16-positive CIN3 lesions. In the analysis including only HPV16-infected samples, the OR was 0.43 for every 10-year increase in age (95% CI: 0.30–0.63).

## 4. Discussion

The main finding of the study is that HPV 16/18-related CIN3 is typical of young women and shows a significant decrease with increasing age. In contrast, it is interesting to note that non-16/18 HPV genotypes become increasingly prevalent with advancing age. Notably, the proportion of CIN3 associated with HPV16 decreased from approximately 85.1% among women <30 years of age to 44.4% among women aged >45 years. There is a significant association between HPV16 positivity and younger age upon CIN3 diagnosis (*p* = 0). Furthermore, the logistic regression analysis highlighted that young age was strongly associated with HPV16 positivity in CIN3 lesions. Specifically, for each 10-year increase, the OR for having CIN3-positive HPV16 was 0.43. The data from the previous study support the results of a series of prospective studies that have highlighted how in many young women, HPV 16 infection can occur immediately after the start of sexual intercourse and lead to the onset of CIN3 within a short while [17,18]. Winer et al. reported the onset of CIN3 within two years of HPV detection [4]. Other authors have found a younger average age at diagnosis for HPV 16/18-positive cervical cancers compared to non-16/18 ones [12,19]. Castle et al. found a 28.8% cumulative risk for CIN3 among persistent HPV 16/18 women and 7.1% for women with other genotypes [20]. The reason why there is a significant correlation between genotypes 16/18 and CIN3 in young women is linked to the oncogenic potential of genotype 16; HPV16 and HPV18 are the most oncogenic [5,21]. Therefore, these infections can rapidly progress to CIN3. In particular, genotype 16 has great oncogenic power; with its ability to evade immune surveillance (HPV16 E5 oncoprotein), it is the most persistent genotype compared to the other high-risk genotypes [22]. In particular, persistent HPV 16 infection is known as the most significant independent prognostic factor in the progression of cervical lesions [23,24]. A previous study [25] showed that the presence of the HPV16 genotype was associated with a five-fold increased risk of developing a high-grade lesion (OR = 4.62 95 CI: 3.13–6.82). It appears that these abilities depend on possessing viral oncogenes (E6/E7) that are more active than other high-risk genotypes. Genotype 16 is the most widespread of all, in all histological grades but also in women with negative cytology [26]. HPV 16 alone is responsible for just over 50% of cervical cancers worldwide. The data from the present study also confirm the frequency and rapidity of the onset of HPV 16-related CIN3, especially in young women. The results of the present study also highlight how CIN3 with non-16/18 HPV genotypes develops at a later age. These data are in agreement with previous studies that have shown that with increasing age, a percentage of cervical cancer appears to be associated with non-HPV 16/18 or HPV-negative [27,28,29].

Rositch et al. reported that, with increasing age, most HPV infections were due to viral reactivation rather than new sexual partners [30]. All this would be due to the immunological and hormonal changes typical of advanced age, which could influence the acquisition and reactivation of some HPV infections [31]. The oncogenic potential of non-16/18 genotypes has been less studied because non-16/18 hr HPV genotypes are often tested as a pool (partial genotyping). The use of extended HPV genotyping would highlight the specific non-HPV 16/18 genotypes by exploring the risk of CIN2+ of each individual genotype.

Non-16/18 HPV genotypes, with lower carcinogenic potential, induce a longer latency to high-grade disease [4,19], grow slowly, and, according to Richart’s step-by-step theory of oncogenesis, they go from CIN1 to CIN2 up to CIN3 [32]. This slower progression may provide a greater chance of detecting these lesions at a pre-invasive stage in a screening program. It is also possible that these weaker carcinogens are more dependent on secondary factors, such as smoking [33]. Indeed, non-HPV 16/18-positive tumors evolve into an invasive state through the accumulation of a greater number of genomic alterations than those required for HPV16/18-positive tumors. In fact, they showed that the number of mutations in cancer genes increases with the age of patients [34].

The results of the study highlight a close correlation of CIN3 with the woman’s age and hr HPV genotypes, highlighting two types of CIN3. Most CIN3 16/18 develop rapidly and are typical of young women; those related to non-16/18 genotypes, with decidedly lower oncogenic potential, grow slowly and are typical of older women. The discovery of CIN3 lesions associated with non-HPV 16/18 (HPV31/39/51/52/59) genotypes in the present study confirms that in women over 45, the frequency of specific genotypes is different from that of women of childbearing age; these data would support the use of extended genotyping for this target group of women.

The limitations of the present research are mainly related to the small sample size and its retrospective nature. The retrospective design generates a large amount of incomplete data and does not allow for the collection of data such as the use of oral contraceptive pills, sexual life, and the use of medications that may contribute to viral regression or persistence. Another potential bias could be that the study population coming from a second-level center is a selected population, so the results are not adaptable to a normal population. The strong point of the study is represented by the homogeneous sample, the multicenter nature, and the fact that centers participating in the study used the same technology for genotyping; finally, it used extensive genotyping, which allowed us to identify non-16/18 HPV genotypes typical of older women.

## 5. Conclusions

The data from the present study suggest that the risk of CIN3 is related to the age of the woman and the hr HPV genotype; there are two types of CIN3: a more frequent type, related to HPV16/18, which develops rapidly and in young women, another, related to non-16/18 HPV, which develops later in old age and slowly, through low-grade lesions. 

## Figures and Tables

**Table 1:** Study population according to age, HPV genotype and histology.

		<30 Years	30–44 Years	≥45 Years
CIN1-2 (*n* = 89)	*n*	*n* = 40	*n* = 34	*n* = 15
HPV 16/18	27	14 (35%)	11 (31.4%)	2 (13.3%)
HPV non 16/18	62	26 (65%)	23 (67.6%)	13 (86.6%)
CIN3 (*n* = 319)	*n*	*n* = 94	*n* = 172	*n* = 53
HPV 16/18	235	80 (85.1%)	130 (75.6%)	25 (47.2%)
HPV non 16/18	84	14 (14.9%)	42 (24.4%)	28 (52.8%)

**Table 2:** Women with CIN3 according to age and HPV genotype.

Years	*n*	HPV16/18	Average Age	No 16/18 HPV	Average Age
<30	94	80 (85.1%)	25.5	14 (14.9%)	27.6
30–44	172	130 (75.6%)	36.3	42 (24.4%)	37.4
>45	53	25 (47.2%)	48.3	28 (52.8%)	54.8
	319	235 (73.7%)		84 (26.3%)	

**Table 3:** The prevalence of HPV infections by age in women with CIN3.

			319 CIN3				
HPV Genotype	≤30 Years *n* = 94		30–44 Years *n* = 172		≥45 Years *n* = 53		
	*n*	(%)	*n*	(%)	*n*	(%)	Total
16	78	(88.6)	130	(75.6)	24	(44.4)	232
18	2	(4.5)	0	(0.0)	1	(7.1)	3
31	7	(7.4)	21	(12.2)	8	(15)	36
33	3	(3.2)	7	(4.0)	4	(7.5)	14
35	0	(0.0)	1	(0.6)	2	(3.7)	3
39	0	(0.0)	0	(0.0)	1	(1.9)	1
45	1	(1.0)	2	(1.2)	2	(3.7)	5
51	0	(0.0)	4	(2.3)	1	(1.9)	5
52	2	(2.1)	5	(2.9)	4	(7.5)	11
53	0	(0.0)	0	(0.0)	2	(3.7)	2
56	1	(1.0)	0	(0.0)	1	(1.9)	2
58	0	(0.0)	2	(1.2)	1	(1.9)	3
59	0	(0.0)	0	(0.0)	2	(3.7)	2

**Table 4:** Odds ratio (OR) with 95% confidence intervals (CI) for the effect of age on CIN3.

Age	CIN3 HPV 16/18	OR	CI 95%	*p*
<30 years	80 (85.1%)	10.61	4.48–25.15	0
30–44 years	130 (75.6%)	6.47	2.91–14.38	0
>45 years	25 (47.1%)	5.80	1.19–28.27	0.02

## Data Availability

The data are contained within the article.
